# 197. The Clinical Impact of Early Detection of ESBL-Producing Enterobacterales with PCR-Based Blood Culture Assays

**DOI:** 10.1093/ofid/ofac492.275

**Published:** 2022-12-15

**Authors:** Kai-Ming Chang, Aya Haghamad, Patricia Saunders-Hao, Sumeet Jain, Marcia Epstein, Vincent Streva, Stefan Juretschko, Pranisha Gautam-Goyal

**Affiliations:** Zucker School of Medicine at Hofstra/Northwell, Manhasset, New York; Northwell Health laboratories, Little Neck, New York; North Shore University Hospital, Manhasset, New York; North Shore University Hospital, Manhasset, New York; Donald and Barbara Zucker School of Medicine at Hofstra/Northwell, Manhasset, New York; Northwell Health Laboratories, Queens, NY; Northwell Health laboratories, Little Neck, New York; Zucker School of Medicine at Hofstra/Northwell, Manhasset, New York

## Abstract

**Background:**

Bloodstream infections (BSIs) due to extended spectrum beta-lactamase (ESBL) producing Enterobacterales can cause significant morbidity and mortality. The first-line treatment for these is a carbapenem. ESBL-encoding genes such as bla_ctx-m_ can be detected by ePlex® Blood Culture Identification (BCID) panels by utilizing polymerase chain reaction (PCR)-based techniques. Our primary objective was to assess the impact of BCID on time to appropriate therapy; secondary objectives were to assess the clinical impact on mortality, 30-day readmission, and length of stay (LOS).

**Methods:**

This study was a retrospective multicenter observational study of hospitalized patients with ESBL-producing Enterobacterales positive blood cultures. Patients with ESBLs identified in the final blood culture by conventional methods pre-ePlex® BCID (Jan 1 to Sep 30, 2019) were compared to patients with ESBLs identified by ePlex® BCID (Jan 1 to Sep 30, 2021). Patients who expired or were discharged prior to Gram stain results were excluded. Time to appropriate therapy was defined as the time from Gram stain result to ordering a carbapenem. In-hospital mortality, 30-day readmission rate, and LOS were analyzed for each cohort. T-test and Chi-square test were performed.

Inclusion and exclusion criteria for pre-ePlex BICD (2019) and post-ePlex BCID (2021) groups

**Figure:**
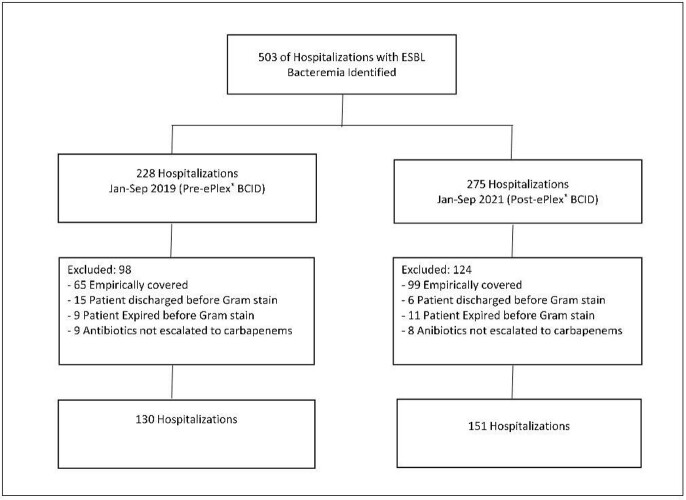

**Results:**

ESBL BSIs in 503 patients across 11 hospitals were identified and a total of 281 patients (pre-ePlex® BCID n=130; ePlex® BCID n=151) were included. A significant decrease in the median time to carbapenem order was observed (44.5 hours in the pre-ePlex® BCID group vs. 8 hours in the ePlex® BCID group (p< 0.001)). No significant difference was observed in in-hospital mortality, 30-day readmissions, or LOS. The mortality rates were 6.9% and 6.6%, the 30-day readmission rates were 7.7% and 12.6%, and the LOS were 8 days and 10 days, respectively in the pre- and post-ePlex® BCID groups.

Comparison of outcome measure results between the pre-ePlex BICD (2019) and post-ePlex BCID (2021) groups.

**Figure:**
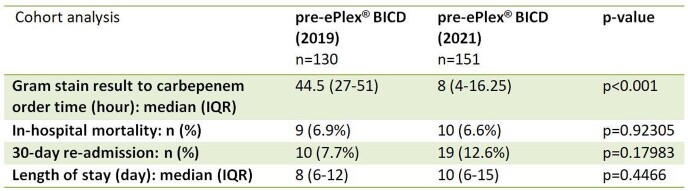

Box and whisker graphs for time from Gram stain result to ordering carbapenems (hours)

**Figure:**
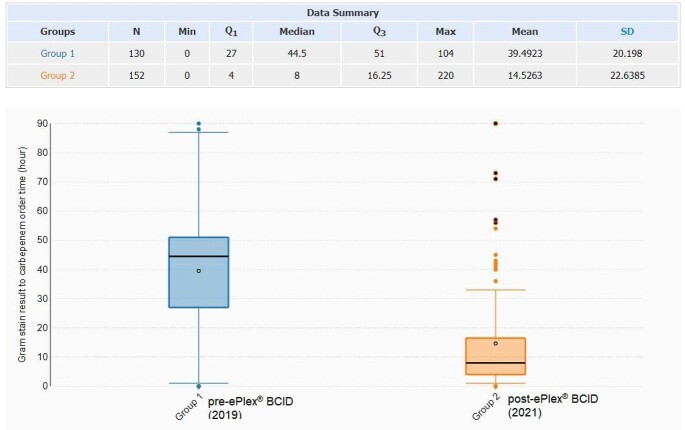

**Conclusion:**

Implementing ePlex® BCID to detect ESBL BSIs decreased the median time to escalation to carbapenems by 36 hours. Despite the decrease in time to appropriate therapy, no change in in-hospital mortality, 30-day readmission, and LOS was observed.

**Disclosures:**

**All Authors**: No reported disclosures.

